# Construction of a comprehensive protein–protein interaction network to identify key genes associated with osteoarthritis

**DOI:** 10.1097/MD.0000000000044534

**Published:** 2025-09-26

**Authors:** Shengcong Guo, Wei Huang, Xiaorong Lu, Li Pang, Jun Yao

**Affiliations:** a Bone and Joint Surgery, The First Affiliated Hospital of Guangxi Medical University, Nanning, China; b Emergency Medicine Department, The Fourth Affiliated Hospital of Guangxi Medical University, Liuzhou, China; c Guangxi Hospital Division of The First Affiliated Hospital, Sun Yat-Sen University, Guangzhou, China.

**Keywords:** autophagy, bioinformatics, diagnostic biomarkers, osteoarthritis, pyroptosis

## Abstract

This research aims to identify key genes and therapeutic targets for osteoarthritis (OA) through bioinformatics, addressing the condition’s significant impact on patients’ lives and healthcare systems. Current treatments are often ineffective, underscoring the need for a deeper understanding of OA’s molecular mechanisms. We investigate cell death mechanisms like pyroptosis and autophagy in OA, using gene expression omnibus datasets to identify differentially expressed genes and develop a protein–protein interaction network highlighting 7 critical genes, including gap junction alpha-1 protein (GJA1) as a potential biomarker. We also created a diagnostic model validated through receiver operating characteristic analysis, which could enhance OA detection and improve patient outcomes. Experimental results from quantitative real-time polymerase chain reaction confirmed the downregulation of GJA1 in the OA group. Significant downregulation of GJA1 expression in patients diagnosed with OA was confirmed. GJA1 may function as a novel regulatory factor in the onset and progression of OA, with potential applications as a diagnostic biomarker for the condition.

## 1. Introduction

Osteoarthritis (OA) is a progressive joint disorder characterized by the slow deterioration of articular cartilage, alterations in the beneath bone structure, and inflammation of the synovial membrane.^[1]^ This condition greatly impacts the quality of life for individuals experiencing it and imposes a substantial financial burden on healthcare systems. The likelihood of developing OA increases with age, making it a critical public health concern worldwide, especially as the population continues to age.^[2]^ Current treatments for OA mainly focus on alleviating symptoms. This highlights the urgent need for a better understanding of molecular mechanisms which involved in the development of OA,^[3]^ which could lead to the creation of more effective treatment options.^[4]^

Previous research has shed light on several pathophysiological factors that contribute to OA, including inflammation, oxidative stress, and changes in cellular metabolism.^[5]^ Among the critical areas of investigation are mechanisms of cell death, particularly pyroptosis and autophagy.^[6]^ Pyroptosis, a type of programmed cell death characterized by inflammation and mediated by gasdermin proteins, is associated with a range of inflammatory conditions, such as OA.^[7]^ This type of cell death leads to the release of pro-inflammatory cytokines, which can worsen joint inflammation and cause further tissue damage.^[8]^ On the other hand, autophagy has a complex role in OA^[9]^; it can support cellular survival and help maintain homeostasis, but when dysregulated, it may contribute to cartilage degradation and inflammation. Understanding how these cellular pathways interact in the context of OA is crucial for unraveling its complex pathophysiology.^[10]^

Our research methodology includes extracting relevant OA datasets from the gene expression omnibus (GEO) database and applying statistical analyses to identify differentially expressed genes (DEGs), which will aid in furthering our understanding of the diseases.

## 2. Materials and methods

### 2.1. Patients samples

All participants consented, and sample procedures followed the Ethics Committee of First Affiliated Hospital of Guangxi Medical University (Approval Number: 2025-E0350). Articular cartilage specimens were obtained through total knee arthroplasty procedures. These samples were selectively harvested from 2 distinct regions of the surgical specimens: severely damaged cartilage regions (outerbridge grades III–IV) and adjacent relatively preserved cartilage regions (outerbridge grades 0–I) in 10 OA patients undergoing joint replacement (5 males and 5 females).

### 2.2. RNA extraction and qRT-PCR

A total of approximately 100 grams of articular cartilage was obtained from each patient for the extraction of total RNA, utilizing the RNAeasy Plus Animal RNA Isolation Kit with Spin Column (Beyotime, Shanghai, China). Subsequently, 1 μg of the extracted RNA was subjected to reverse transcription into complementary DNA using the PrimeScript RT reagent kit with gDNA Eraser (TaKaRa, Dalian, China). To evaluate the expression levels of gap junction alpha-1 protein (GJA1) messenger RNA (mRNA) in both regions of severely damaged cartilage and in the adjacent areas of relatively preserved cartilage, quantitative real-time polymerase chain reaction (qRT-PCR) was employed. The primers for qRT-PCR were obtained from sequences available in the GenBank database. Each reaction mixture was composed of 0.5 μL of each primer, 1.5 μL of sterile distilled water, 2.5 μL of complementary DNA, and 5 μL of SYBR Premix Ex Taq mix (Thermo Fisher Scientific, Waltham), resulting in a total reaction volume of 10 μL. For normalization purposes, GAPDH was used, and comparative analysis with control samples was performed utilizing the 2^−ΔΔCt^ method. To ensure the reliability of the results, each experiment was conducted in triplicate. The primer sequences utilized in this study were as follows:

GJA1: Forward: 5′-TCTGAGTGCCTGAACTTGCC-3′;

Reverse: 5′-GCCTGGGCACCACTCTTTTG-3′.

GAPDH: Forward: 5′-CACCCACTCCTCCACCTTTGAC-3′.

Reverse: 5′-GTCCACCACCCTGTTGCTGTAG-3′.

### 2.3. Data source

We sourced the GSE98918 dataset from GEO using “Osteoarthritis,” filtering it to align with our study. The dataset includes 12 OA samples and 12 from meniscectomy, forming our training dataset. We also selected GSE51588, which has 20 OA and 5 control samples for external validation, and GSE169077 with 5 OA and 6 control samples for further validation. Additionally, we obtained autophagy-related and pyroptosis-related genes from GeneCards and the Gene Set Enrichment Analysis (GSEA)/Molecular Signatures Database.

### 2.4. Screening DEGS and autophagy and pyroptosis-related differentially expressed genes (APRDEGs)

The transcriptome dataset GSE98918 was analyzed using “limma” R package (version 3.52.2, Australia Department of Mathematics and Statistics, The University of Melbourne, Melbourne, Australia). DEGs were identified with a threshold for adjusted *P* values set below .05 and a |log fold change (FC)| exceeding 1. To illustrate the DEGs, both heatmaps and volcano plots were utilized. A Venn diagram demonstrated the overlap of DEGs with antimicrobial resistance genes (ARGs) and prognostic-related genes (PRGs), highlighting the APRDEGs.

### 2.5. Gene enrichment analysis

Kyoto Encyclopedia of Genes and Genomes (KEGG) and Gene Ontology (GO) pathway enrichment analyses for APRDEGs were performed using “clusterProfiler” package on Xiantao online platform (https://www.xiantaozi.com/, version 2.0). The analysis of GO functional enrichment included evaluations of terms related to molecular function, cellular component, and biological process (BP). A gene set was classified as significantly enriched when *P*.adj was <.05. To explore the possible BPs linked to genes associated with OA, GSEA was conducted using “clusterProfiler” R package for the evaluation. Significant enrichment was considered when *P*-values were under .05 and false discovery rate had a *q*-value of <0.25.

### 2.6. Identification of hub genes and investigation of intermolecular interactions of APRDEGs

To enable a deeper investigation into the functions of autophagy and pyroptosis in OA, the STRING database (https://string-db.org/, version 12.0) was used to build a protein–protein interaction (PPI) network for APRDEGs. Following this, the cytoHubba tool available in the Cytoscape (version 3.10.0, https://cytoscape.org/) was applied to identify the hub genes present in the created PPI network.

### 2.7. Expression of hub genes in dataset GSE98918 and validation using external data

Violin plots were generated to evaluate the expression differences of hub genes between OA group and control group. To reduce the likelihood of false positives, the expression levels of the hub genes were verified using an independent dataset obtained from GEO database. The GSE51588 dataset was selected for this validation, which includes data from 20 patients with OA and 5 controls. Subsequently, the expression data for the hub genes were collected and depicted through violin plots to facilitate comparative analysis.

### 2.8. Analysis of immune infiltration and correlations between hub genes and immune cells

The single-sample Gene Set Enrichment Analysis (ssGSEA) algorithm provides an advanced computational framework intended to convert normalized gene expression data into comprehensive profiles that illustrate immune cell infiltration. Utilizing leukocyte mixture 24 reference expression signature, we meticulously calculated the relative frequencies of various immune cell types, distinguishing between OA patients and the control group, aided by the “GSVA[1.44.5]” R package (version 4.2.1). To elucidate the relationships between immune cells and crucial hub genes, we performed a comprehensive Pearson correlation analysis. The processed data were systematically organized and visually represented through both violin and heat maps, utilizing an online platform (https://www.xiantaozi.com/, version 2.0).

### 2.9. Validation and screening of potential diagnostic biomarkers

To enhance the understanding of the clinical significance of the hub genes, we constructed receiver operating characteristic (ROC) curves to assess the potential diagnostic value of these genes based on area under curve (AUC). The ROC curves were developed using mRNA expression data obtained from GSE98918 dataset, and the results were further validated in GSE51588 and GSE169077 dataset. A statistical evaluation threshold of *P* <.05 was set for significance.

## 3. Results

### 3.1. Identification of DEGs and the landscape of APRDEGs

**Figure 1.**
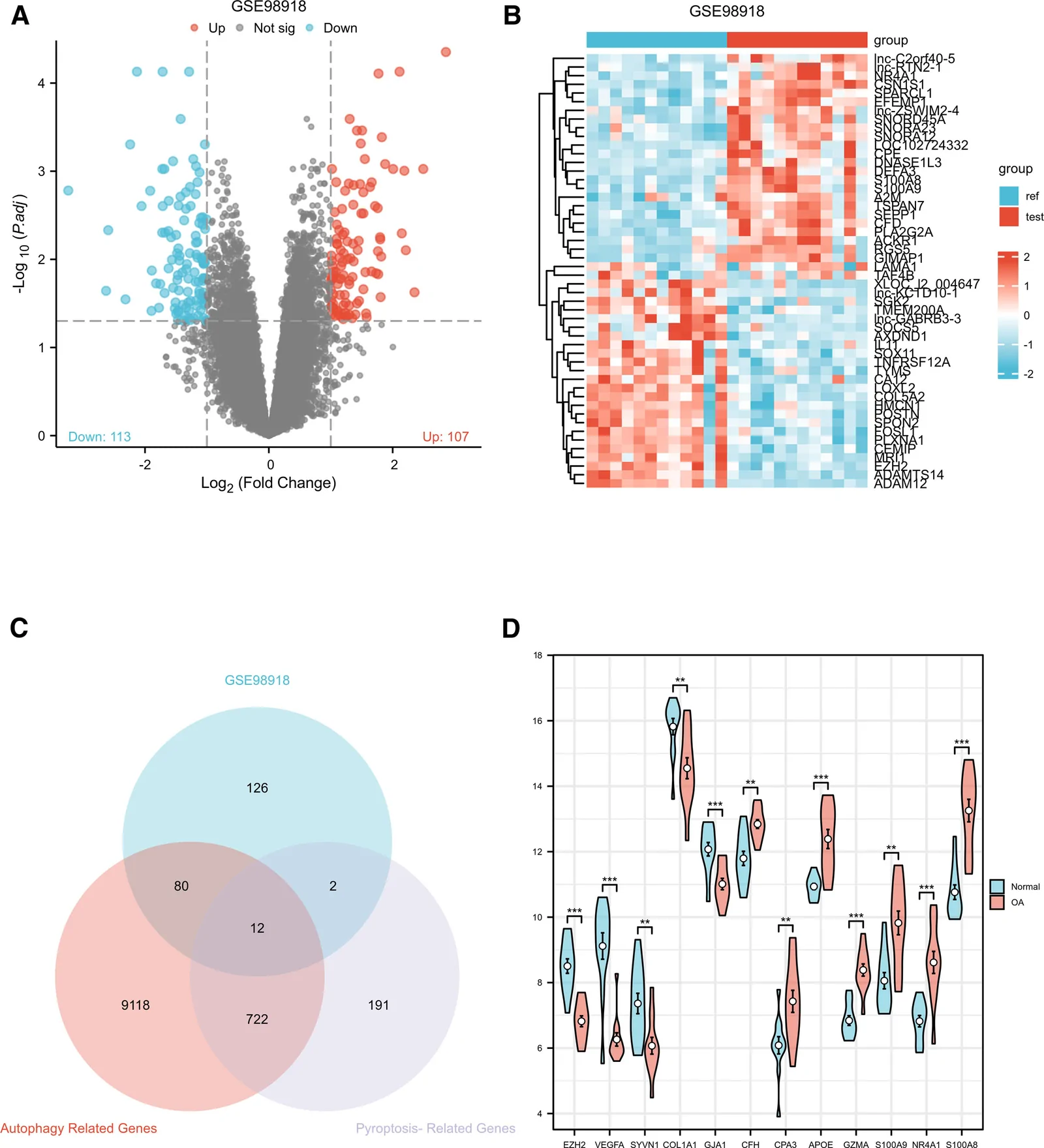
Identification and expression analysis of differentially expressed genes (DEGs) and apoptosis-related differentially expressed genes (APRDEGs) in osteoarthritis. (A) Bar graph showing the total number of DEGs identified from the GSE98918 microarray dataset. A total of 220 DEGs were identified, including 107 up-regulated and 113 down-regulated genes. (B) Heatmap depicting the expression profile of the identified DEGs, highlighting the significant differences between osteoarthritis patients and healthy controls. (C) Venn diagram illustrating the intersection of DEGs with apoptosis-related genes (ARGs) and programmed cell death-related genes (PRGs), resulting in the identification of 12 APRDEGs. (D) The list of identified APRDEGs, which include enhancer of zeste homolog 2 (EZH2), vascular endothelial growth factor A (VEGFA), synovial apoptosis inhibitor 1 (SYVN1), collagen type I alpha 1 chain (COL1A1), complement factor H (CFH), carboxypeptidase A3 (CPA3), apolipoprotein E (APOE), granzyme A (GZMA), S100 calcium binding protein A9 (S100A9), nuclear receptor subfamily 4 group A member 1 (NR4A1), and GJA1, showing significant expression differences between osteoarthritis patients and healthy controls. GJA1 = gap junction alpha-1 protein.

To clarify the pathways and biological mechanisms linked to OA, we identified a total of 220 DEGs from GSE98918 microarray dataset, following the selection criteria previously outlined. Among them, 107 were up-regulated, while 113 were down-regulated, as illustrated in Figure 1A and B. Additionally, we screened 9932 ARGs and 927 PRGs. By intersecting the DEGs with the ARGs and PRGs, we identified 12 APRDEGs, which showed significant expression differences between OA patients and healthy controls, as shown in Figure 1D. The APRDEGs identified in this study include enhancer of zeste homolog 2, vascular endothelial growth factor A, synovial apoptosis inhibitor 1, collagen type I alpha 1 chain (COL1A1), complement factor H, carboxypeptidase A3, apolipoprotein E, granzyme A, S100 calcium binding protein A9, nuclear receptor subfamily 4 group A member 1, and GJA1.

### 3.2. Functional enrichment analysis

**Figure 2.**
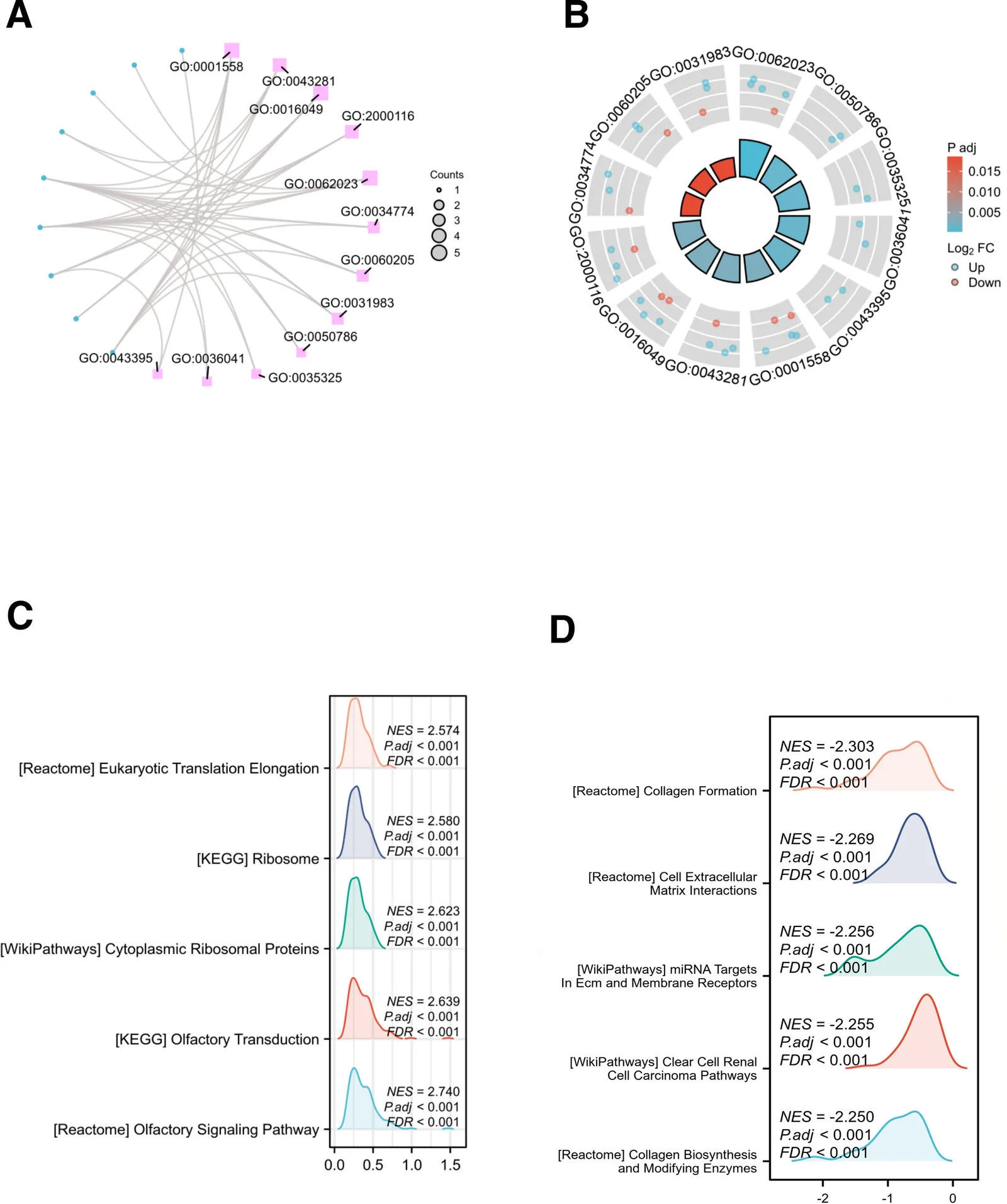
Functional enrichment analysis of apoptosis-related differentially expressed genes (APRDEGs) in osteoarthritis. (A) Gene ontology (GO) enrichment analysis results of APRDEGs, highlighting significant biological processes, including (1) control of cell proliferation (GO: 0001558), (2) modulation of cysteine-type endopeptidase activity associated with apoptosis (GO: 0043281), and (3) overall cell growth (GO: 0016049). Additional enriched biological processes are also noted. Significant molecular functions include: (1) receptor for advanced glycation endproducts (RAGE) receptor binding (GO: 0050786), (2) toll-like receptor binding (GO: 0035325), and (3) long-chain fatty acid binding (GO: 0036041). (B) FC-string diagram illustrating the combined GO–KEGG analysis results, revealing potential pathways associated with osteoarthritis. (C, D) Gene Set Enrichment Analysis (GSEA) results showing the enriched pathways in the context of arthritis, including the reactome olfactory signaling pathway, KEGG olfactory transduction, and Whitehead Proteome Collection (WPC) cytoplasmic ribosomal proteins. The normalized enrichment score (NES) values for the top 5 and bottom 5 pathway IDs are graphically represented, providing insight into the pathways most significantly associated with osteoarthritis. GO = Gene Ontology, KEGG = Kyoto Encyclopedia of Genes and Genomes.

To analyze the functions of APRDEGs, we conducted GO and KEGG enrichment analyses, we began by exploring the biological characteristics of APRDEGs through GO annotation. The main BPs recognized encompassed the control of cell proliferation (GO: 0001558), the modulation of cysteine-type endopeptidase activity associated with apoptosis (GO: 0043281), and overall cell growth (GO: 0016049), among additional factors. In terms of molecular functions, we observed significant enrichment for receptor for advanced glycation endproducts receptor binding (GO: 0050786), toll-like receptor binding (GO:0035325), and long-chain fatty acid binding (GO:0036041), as illustrated in Figure 2A. Furthermore, we performed a combined GO–KEGG analysis, which included a FC-String diagram depicted in Figure 2B. This comprehensive approach allowed us to delve deeper into the potential pathways associated with OA. In the context of arthritis, GSEA was performed, highlighting that the enriched pathways predominantly included the reactome olfactory signaling pathway, KEGG olfactory transduction, and whitehead proteome collection cytoplasmic ribosomal proteins, among others. By examining the normalized enrichment score values, we identified the top 5 and bottom 5 pathway IDs, with the graphical representation of these results displayed in Figure 2C and D.

### 3.3. PPI network analysis for identifying hub genes and interactions among hub genes

**Figure 3.**
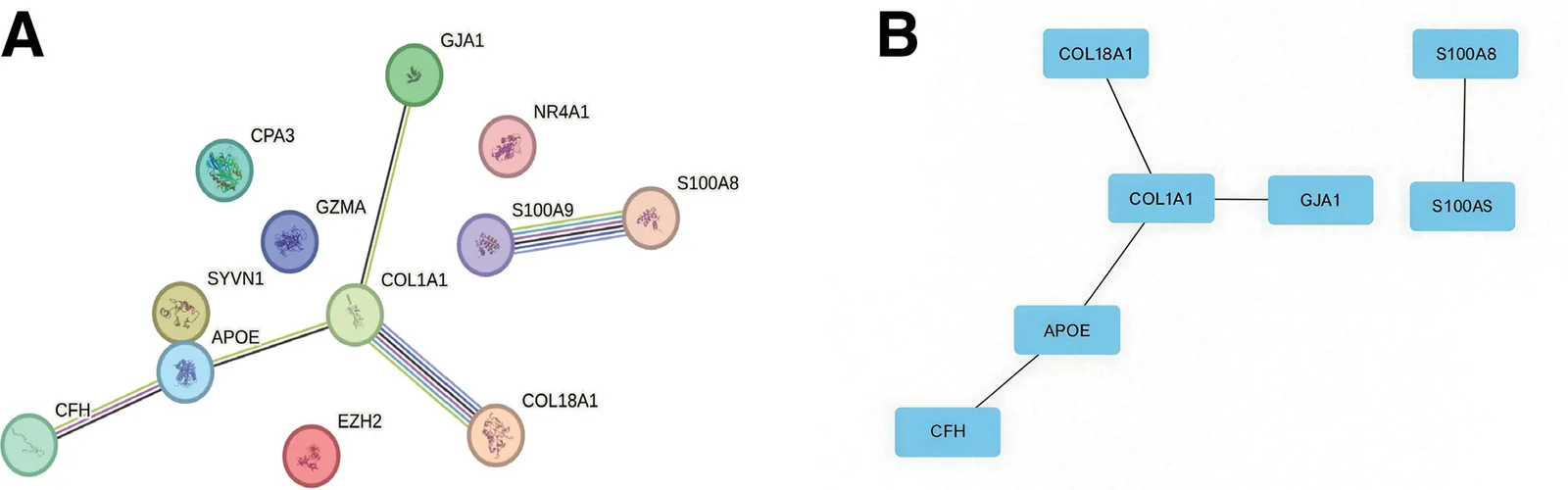
Protein–protein interaction (PPI) network analysis for identifying hub genes in osteoarthritis. (A) PPI network developed using the STRING online database, focusing on the differentially expressed genes (DEGs) related to osteoarthritis. The network illustrates interactions among DEGs, highlighting potential biological relationships. (B) Identification of hub genes using the cytoHubba plugin in Cytoscape. The hub genes identified in this study include collagen type I alpha 1 chain (COL1A1), collagen type XVIII alpha 1 chain (COL18A1), GJA1, complement factor H (CFH), apolipoprotein E (APOE), S100 calcium binding protein A8 (S100A8), and S100 calcium binding protein A9 (S100A9). The visual representation emphasizes their central roles in the PPI network.

To identify hub genes and their interactions in OA, we developed a PPI network using the STRING online database, focusing on DEGs. This analysis is illustrated in Figure 3A. We identified the hub genes through the cytoHubba plugin in Cytoscape. The hub genes identified in this study include COL1A1, COL18A1, GJA1, complement factor H, apolipoprotein E, S100 calcium binding protein A8, and S100 calcium binding protein A9, as shown in Figure 3B.

### 3.4. Immune infiltration analysis and correlations between hub genes and immune cells

**Figure 4.**
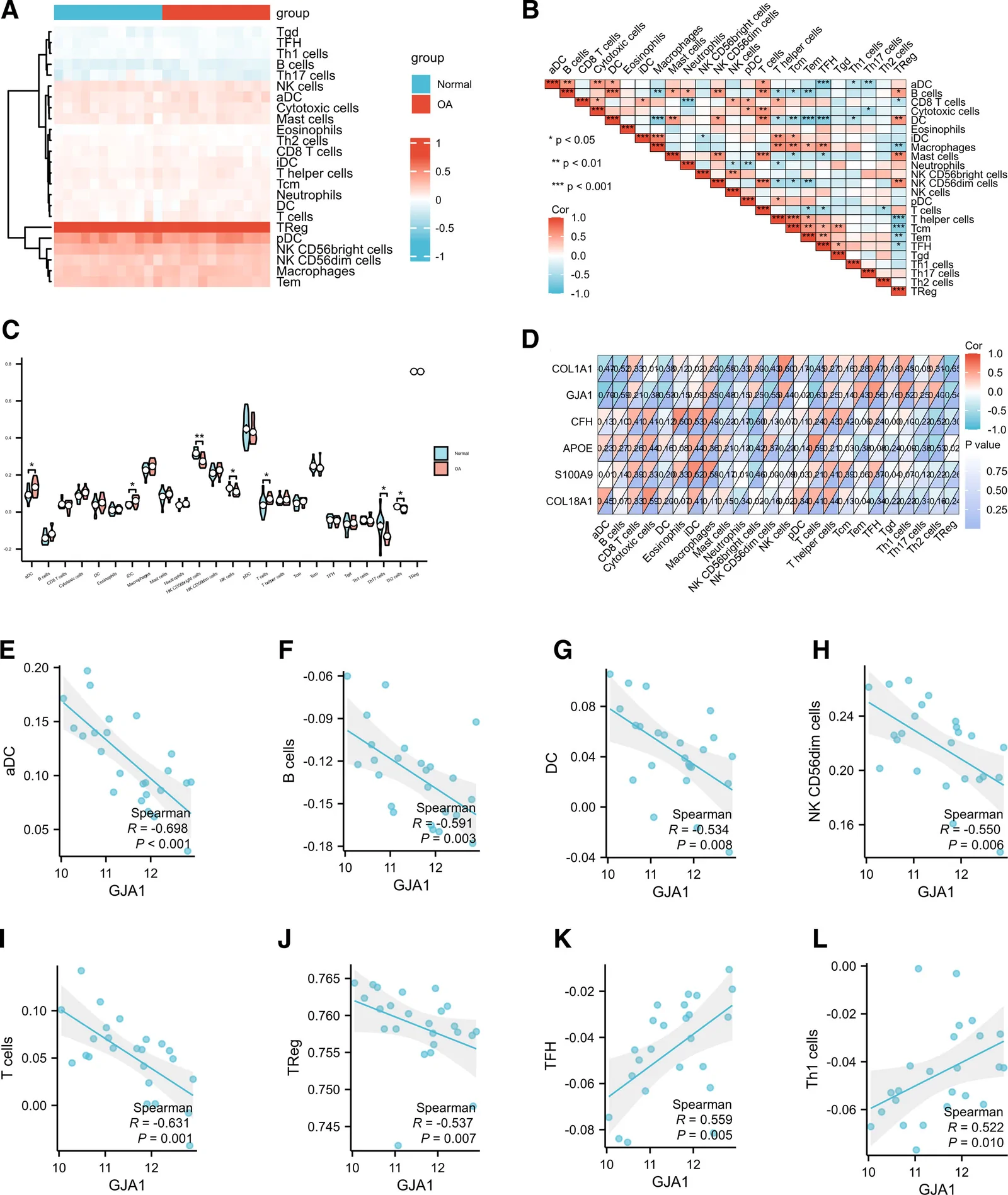
Immune infiltration analysis and correlations with hub genes in osteoarthritis. (A) Heatmap showing the distribution of 24 immune cell types across 12 osteoarthritis samples and 12 arthroscopic partial meniscectomy samples. (B) Significant positive correlations between memory resting dendritic cells and B cells, as well as T cells and mast cells (*P* < .001), and negative correlations between TFH cells and aDCs/DCs (*P* < .001). (C) Mountain range diagram revealing differences in immune cell infiltration, with aDCs, iDCs, NK cells, T cells, and TH2 cells increasing in osteoarthritis patients, while NK CD56 bright and TH17 cells decreased (*P* < .05). (D) Correlation analysis showing significant negative correlations between GJA1 and aDCs, B cells, DCs, NK CD56 dim cells, T cells, and TReg cells, while positive correlations were found with TFH and TH1 cells. (E–L) The results demonstrate the correlation between various immune cell types and GJA1, showing that multiple cell populations (such as aDC cells, B cells, and T cells) exhibit negative correlations, while TFH and TH1 cells display positive correlations with GJA1. aDC = activated dendritic cells, DC = dendritic cells, GJA1 = gap junction alpha-1 protein, iDCs = immature DC cells, TFH = T follicular helper.

An immune infiltration analysis was performed using single-sample gene set enrichment analysis inverse convolution method, comparing 12 OA samples with 12 samples from arthroscopic partial meniscectomy, as depicted in the heat map. The distribution of 24 different types of immune cells across these samples is illustrated in Figure 4A. A significant positive correlation was found between memory resting dendritic cells (DC) and B cells (*P* < .001), as well as between T cells and mast cells (*P* < .001) (Fig. 4B). Conversely, a negative correlation was noted between T follicular helper (TFH) cells and activated dendritic cells (aDC) (*P* < .001), along with a negative correlation between TFH cells and DC cells (*P* < .001) (Fig. 4B). The differences in immune cell infiltration were represented by a mountain range diagram that contrasted the tissues of healthy individuals with those of OA patients, revealing statistically significant results at *P* < .05. Specifically, aDC cells, immature DC cells, NK cells, T cells, and TH2 cells exhibited a differential increase in OA patients, while NK CD56 bright cells and TH17 cells were found to be differentially reduced (Fig. 4C). The correlations between key APRDEGs and immune-infiltrated cells in OA, which were distinct between OA patients and healthy controls, were evaluated using Spearman correlation analysis (Fig. 4D). A significant negative correlation was established between aDC cells and GJA1 (*r* = −0.698, *P* < .001). Similarly, B cells (*r* = −0.591, *P* = .003), DC cells (*r* = −0.534, *P* = .008), NK CD56 dim cells (*r* = −0.550, *P* = .006), T cells (*r* = −0.631, *P* = .001), and regulatory T cells (*r* = −0.537, *P* = .007) also exhibited negative correlations with GJA1. In contrast, TFH cells (*R* = 0.559, *P* = .005) and TH1 cells (*R* = 0.522, *P* = .010) showed positive correlations with GJA1 (Fig. 4E–L).

### 3.5. GJA1 has potential diagnostic power

**Figure 5.**
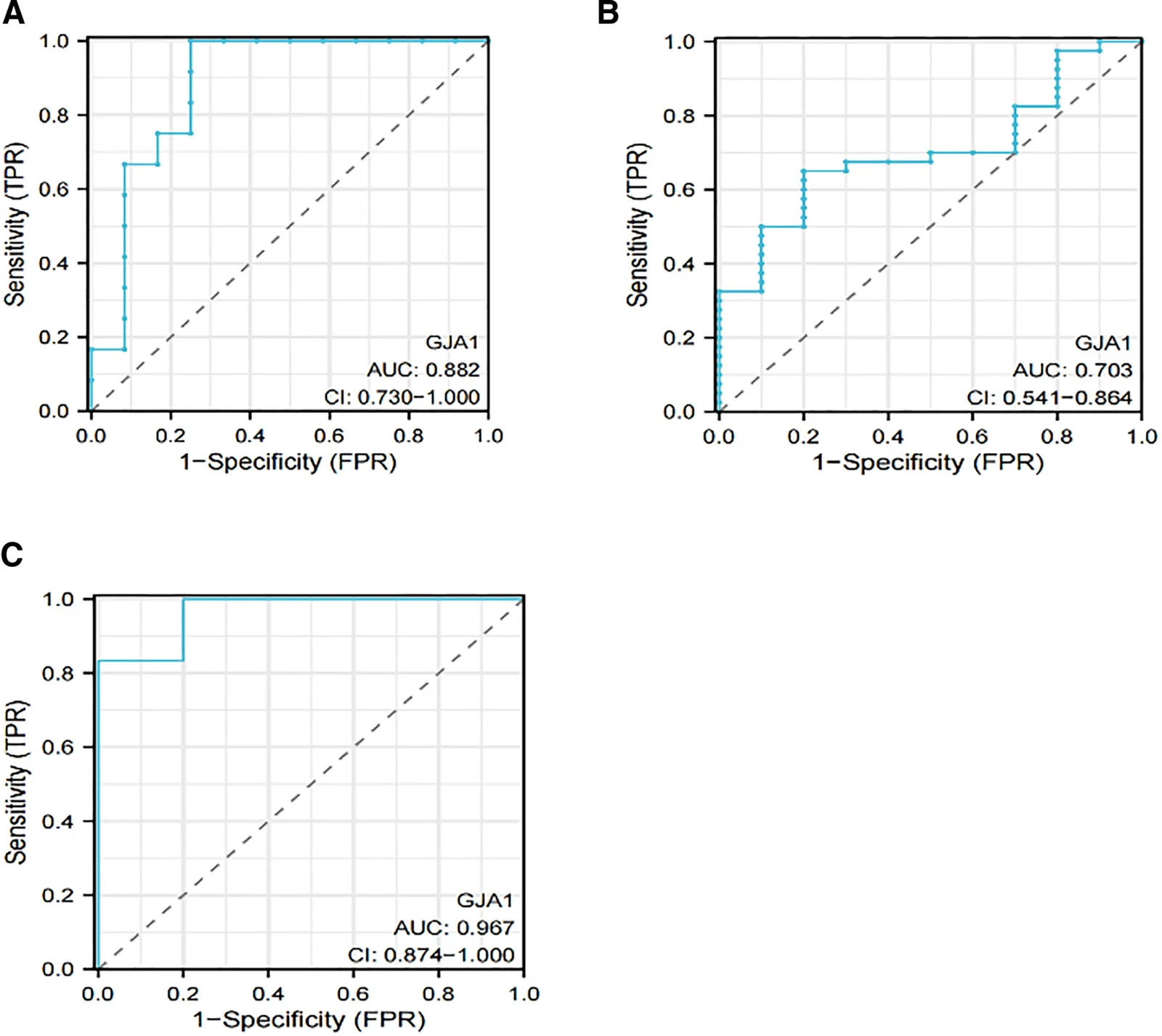
Diagnostic potential of GJA1 in osteoarthritis. (A) ROC curve for GJA1 in the primary cohort, with an AUC of 0.882 (95% CI: 0.730–1.000). (B) ROC curve for GJA1 in the GSE51588 cohort, AUC of 0.703 (95% CI: 0.541–0.864). (C) ROC curve for GJA1 in the GSE169077 cohort, AUC of 0.967 (95% CI: 0.874–1.000). These results suggest GJA1’s significant role in osteoarthritis pathology. AUC = area under curve, GJA1 = gap junction alpha-1 protein, ROC = receiver operating characteristic.

To systematically evaluate the diagnostic potential of GJA1 identified in prior analyses, we constructed ROC curves and calculated corresponding AUC values across multiple OA datasets. As demonstrated in Figure 5A, GJA1 exhibited robust diagnostic performance in the primary cohort, achieving an AUC of 0.882 (95% CI: 0.730–1.000). Notably, this diagnostic capacity remained consistent in independent validation cohorts: GJA1 yielded an AUC of 0.703 (95% CI: 0.541–0.864) in the GSE51588 dataset (Fig. 5B), while reaching a remarkably high AUC of 0.967 (95% CI: 0.874–1.000) in the GSE169077 cohort (Fig. 5C). These findings imply that GJA1 may have a significant role in pathological mechanisms underlying OA.

### 3.6. GJA1 expression verification

**Figure 6.**
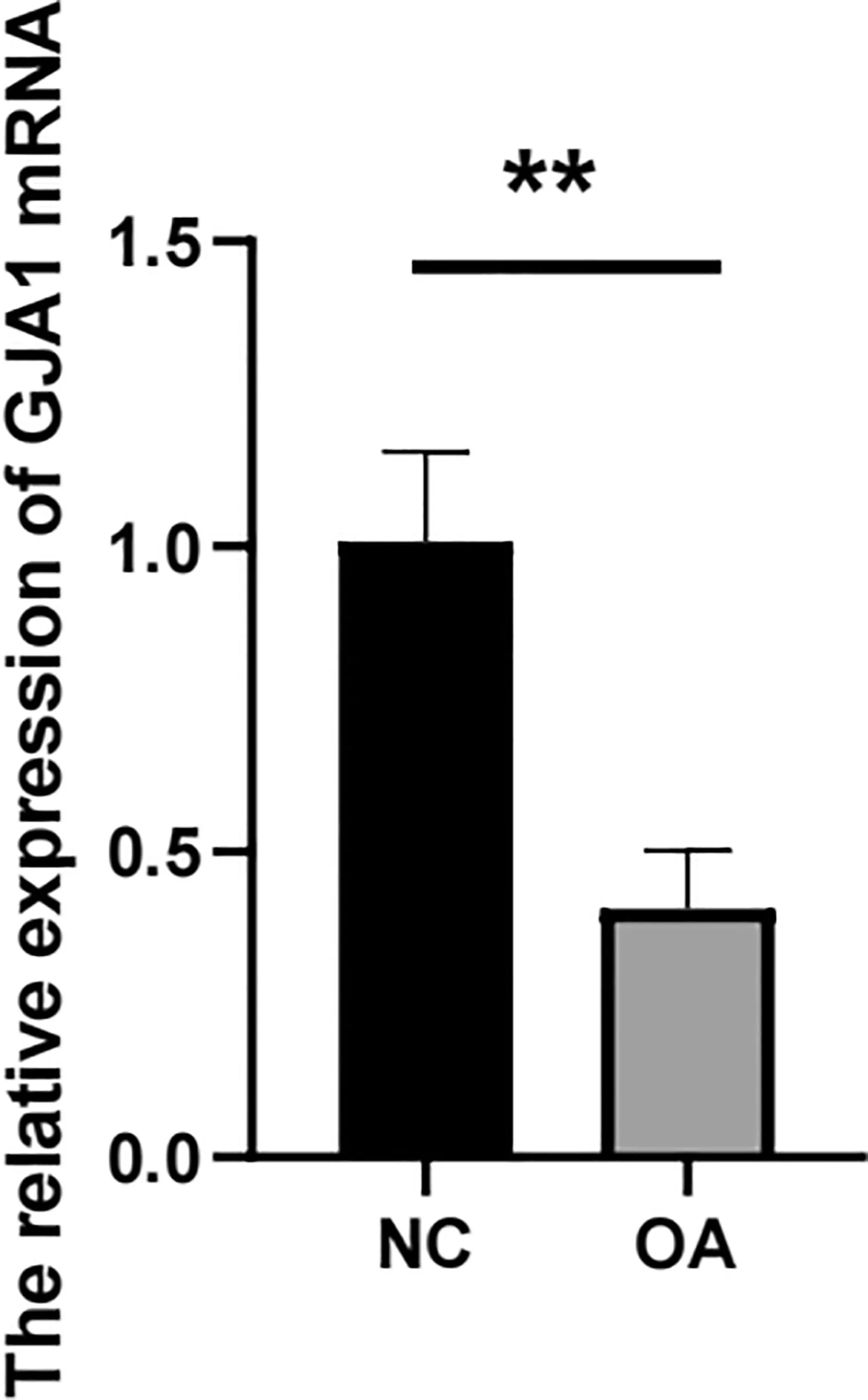
Verification of GJA1 expression in osteoarthritis. Bioinformatics analysis revealed significantly reduced GJA1 expression in osteoarthritis (OA) chondrocytes compared to normal chondrocytes (*P* < .01). qRT-PCR analysis confirmed markedly lower GJA1 mRNA levels in the OA group relative to the normal group. GJA1 = gap junction alpha-1 protein, qRT-PCR = quantitative real-time polymerase chain reaction. ** represent the *P* values < .01.

Bioinformatics investigation showed significantly reduced GJA1 expression in OA group chondrocytes compared to normal group chondrocytes (*P* < .01). This finding was further corroborated by qRT-PCR analysis, which demonstrated markedly lower levels of GJA1 mRNA expression in the OA group compared to the normal group (Fig. 6).

## 4. Discussion

OA is a prevalent and complex joint disorder characterized by the gradual breakdown of cartilage, alterations in bone structure, and the formation of osteophytes, which collectively lead to significant pain and functional limitations that can severely impact an individual’s quality of life while also placing considerable strain on healthcare systems.^[11,12]^ The etiology of OA is multifaceted, encompassing a range of contributing factors such as the natural aging process, the mechanical load placed on joints, obesity, and genetic predispositions, all of which complicate the development of effective therapeutic interventions.^[13,14]^ Current treatment modalities primarily aim to alleviate symptoms rather than addressing the underlying pathological processes, underscoring the urgent need for a more profound molecular understanding of the disease.^[15]^ Acknowledging the critical role that cartilage plays in the progression of OA, our research specifically investigates how alterations in chondrocyte phenotype can initiate the onset of OA, occurring independently of subsequent changes in the synovial membrane or bone structure, thereby providing new insights into the early mechanisms of this debilitating condition.^[16,17]^

Our research specifically explores the roles of pyroptosis and autophagy in OA pathology. Utilizing data from the GEO, we identified DEGs that illuminate biological pathways integral to OA progression. Notably, 7 crucial genes were pinpointed, with GJA1 emerging as a prospective diagnostic biomarker for OA.

Through the development of a PPI network, we identified 7 key genes (including COL1A1, COL18A1, and GJA1) that are central to OA pathophysiology. Of these genes, COL1A1 has been previously linked to the crucial maintenance of cartilage integrity and the complex inflammatory processes that are associated with OA, a degenerative joint disease that affects millions worldwide.^[18]^ Specifically, detailed studies conducted on murine models have corroborated that the overexpression of COL1A1 significantly drives the transition of fibrocartilage through the extensive deposition of type I collagen, which, in turn, disrupts the delicate biomechanics of cartilage, leading to further complications in joint function and health.^[19]^ In contrast, COL18A1 interacts intricately with various growth factors and cytokines, playing a crucial role in the modulation of tissue remodeling processes; however, its function is fundamentally distinct from that of COL1A1. Specifically, while COL18A1 works in concert with type IV collagen to provide essential stabilization to the basement membrane,^[20]^ its deficiency may lead to mechanical instability at the cartilage-bone junction. This instability occurs without directly inducing the degradation of cartilage, highlighting a unique aspect of COL18A1’s role in maintaining structural integrity within the tissue architecture. Notably, previous studies have shown that the genes GJA1 and COL1A1 exhibit a remarkable functional complementarity in the intricate process of early cartilage formation,^[21]^ suggesting that their interactions play a crucial role in the development and structural integrity of cartilage tissue during the initial stages of skeletal development. Moreover, GJA1 exhibits a dual regulatory mechanism absent in other OA-associated genes. Unlike the unidirectional role of COMP in the degradation of the extracellular matrix, which functions in a straightforward and singular manner, GJA1 reduces the phosphorylation levels of ERK1/2 by inhibiting the activity of the MAPK/ERK signaling pathway, thereby decreasing inflammasome-mediated pyroptosis and promoting the expression of autophagy-related proteins, maintaining the dynamic balance between pyroptosis and autophagy.^[22]^ On one hand, it inhibits NLRP3 inflammasome activation and downstream gasdermin D-mediated membrane pore formation,^[23]^ thereby potentially reducing the release of pyroptosis-related factors (IL-1β, IL-18) in chondrocytes. Simultaneously, it enhances autophagic flux by activating the ULK1-Beclin1 complex, promoting the clearance of damaged mitochondria and protein aggregates. This bidirectional regulatory mechanism effectively maintains chondrocyte homeostasis, counteracting the characteristic “pyroptosis–autophagy imbalance” vicious cycle in OA, where excessive pyroptosis releases matrix metalloproteinases (MMPs) that accelerate articular extracellular matrix (ECM) degradation,^[24]^ while autophagic deficiency results in abnormal protein accumulation that induces endoplasmic reticulum stress.^[25]^

While COMP,^[26]^ or cartilage oligomeric matrix protein, and MMPs^[27,28]^ are well-established markers utilized for the assessment of ECM degradation (where COMP serves as an indicator of cartilage oligomeric matrix protein loss and MMPs reflect the activity of collagenases involved in the breakdown of collagen) GJA1, presents a unique and valuable mechanistic insight. This insight emphasizes the critical role of gap junction-mediated intercellular communication and highlights the early cellular stress responses occurring within chondrocytes.^[29]^ Unlike COMP and MMPs, which are primarily associated with the later stages of ECM degradation,^[30–32]^ GJA1 may serve as an early warning signal for pathological changes that occur prior to significant ECM breakdown.^[33]^ These changes may include mechanical stress and inflammatory interactions that can affect chondrocyte function. Consequently, GJA1 holds the potential to offer significant diagnostic benefits for the early identification of OA, allowing for timely intervention and management of the disease before it progresses to more advanced stages.

We established a diagnostic framework for OA based on ROC analysis, demonstrating high specificity and sensitivity, offering promise for clinical application. This approach effectively assesses biomarker efficacy, suggesting enhancements in OA detection and intervention. Our results identify GJA1 as a significant OA biomarker, though our study acknowledges inherent limitations.

Despite the successful identification of clinically relevant biomarkers through our bioinformatics methodology, it is essential to recognize that the GEO datasets predominantly comprise samples from patients at advanced OA stages who underwent joint replacement surgeries. This selection bias may obscure the evaluation of early diagnostic opportunities. The molecular profiles captured are likely representative of established pathological alterations rather than the disease’s initial stages. Nonetheless, these molecular signatures offer critical insights into the mechanisms driving disease development.

The hub genes identified, particularly GJA1, are functionally linked to the remodeling of the cartilage matrix and the activation of inflammatory pathways: key actors in OA progression. Although our primary focus has been on the intricate interaction between autophagy and pyroptosis in the context of OA, we fully acknowledge the considerable significance of delving into other regulated cell death pathways that may play a crucial role in this disease. GSEA has revealed that pathways associated with ferroptosis and apoptosis are not only present but are also significantly enriched, suggesting that these mechanisms warrant further investigation. Looking ahead, future multimodal studies that integrate advanced techniques such as single-cell sequencing and comprehensive lipid peroxidation profiling could provide invaluable insights, allowing us to further elucidate the unique contributions and intricate interplay of diverse cell death mechanisms in the progression of OA, thereby enhancing our understanding of this complex condition.

Given the limited sample size, caution is necessary when interpreting the complexity of OA, as it may lead to an underestimation of its multifaceted pathological characteristics.

## 5. Conclusions

In summary, this research has successfully identified GJA1 as a potential diagnostic biomarker for OA and has elucidated the roles of key genes associated with inflammation and autophagy. These results enhance our comprehension of the molecular processes involved in OA and establish a strong basis for upcoming research focused on advancing diagnostic and treatment approaches. The integration of bioinformatics techniques with experimental validation is crucial for refining these discoveries and enhancing their clinical applicability in the management of OA.

## Acknowledgments

We thank everyone in the Bone and Joint Surgery of The First Affiliated Hospital of Guangxi Medical University for all their constructive comments and advice.

## Author contributions

**Data curation:** Li Pang.

**Methodology:** Xiaorong Lu.

**Visualization:** Wei Huang.

**Writing – original draft:** Shengcong Guo.

**Writing – review & editing:** Jun Yao.
